# Unprecedented Disease-Related Coral Mortality in Southeastern Florida

**DOI:** 10.1038/srep31374

**Published:** 2016-08-10

**Authors:** William F. Precht, Brooke E. Gintert, Martha L. Robbart, Ryan Fura, Robert van Woesik

**Affiliations:** 1 Marine and Coastal Programs, Dial Cordy and Associates, Inc. 1001 Ives Dairy Road, Suite 210, Miami, FL 33179, USA; 2 Division of Marine Geosciences, Rosenstiel School of Marine and Atmospheric Science, University of Miami, 4600 Rickenbacker Causeway, Miami, FL 33149, USA; 3 Department of Biological Sciences Florida Institute of Technology, 150 West University Boulevard, Melbourne, FL 32901, USA

## Abstract

Anomalously high water temperatures, associated with climate change, are increasing the global prevalence of coral bleaching, coral diseases, and coral-mortality events. Coral bleaching and disease outbreaks are often inter-related phenomena, since many coral diseases are a consequence of opportunistic pathogens that further compromise thermally stressed colonies. Yet, most coral diseases have low prevalence (<5%), and are not considered contagious. By contrast, we document the impact of an extremely high-prevalence outbreak (61%) of white-plague disease at 14 sites off southeastern Florida. White-plague disease was observed near Virginia Key, Florida, in September 2014, and after 12 months had spread 100 km north and 30 km south. The disease outbreak directly followed a high temperature coral-bleaching event and affected at least 13 coral species. *Eusmilia fastigiata, Meandrina meandrites,* and *Dichocoenia stokesi* were the most heavily impacted coral species, and were reduced to <3% of their initial population densities. A number of other coral species, including *Colpophyllia natans, Pseudodiploria strigosa, Diploria labyrinthiformis,* and *Orbicella annularis* were reduced to <25% of their initial densities. The high prevalence of disease, the number of susceptible species, and the high mortality of corals affected suggests this disease outbreak is arguably one of the most lethal ever recorded on a contemporary coral reef.

Over the last thirty-five years, thermal-stress events have become increasingly severe and frequent on coral reefs^1^,^2^. These thermal-stress events are associated with global climate change and have caused directional selection, removing thermally susceptible species from many coral reefs^3^. Thermal-stress events also have been linked to coral-disease outbreaks, particularly in the Caribbean^4^,^5^, resulting in widespread reductions of *Acropora* spp. populations^5^,^6^. Yet, our understanding of coral epidemiology is in its infancy. We know little about the typical levels of disease prevalence in coral populations, and we know even less about disease incidence. Furthermore, we have little information on whether particular diseases are potentially transmissible to neighboring conspecifics, and whether some diseases are discriminatory across species boundaries. Moreover, if diseases are transmissible, it is important to know under which environmental circumstances, and by which vectors, coral diseases are transmitted^7^. As the oceans continue to warm, it is likely that the impact of coral diseases will continue to increase in both intensity and geographic extent in the near future^8^. It is therefore critical to understand coral epidemiology as a first step in the potential remediation of coral-disease outbreaks.

The first published record of a major coral-disease outbreak was in 1975 on Carysfort Reef, in the upper Florida Keys^9^; Dustan^9^ recorded “plague-like symptoms” on six reef-building species. In 1995, another plague-like outbreak swept through the corals in the upper Florida Keys^10^,^11^,^12^. Richardson *et al*.^10^ coined the term white-plague type-II to distinguish the 1995 outbreak from previous plague-like disease outbreaks. From 1995 to 1997, the white-plague-type-II disease spread both north and south along the Florida reef tract, affecting a total of 17 scleractinian coral species^10^,^11^. The 1995 outbreak particularly impacted populations of *Dichocoenia stokesi,* resulting in 38% mortality^12^. The outbreak diminished during each successive winter, and resumed as the water temperatures increased, in the spring and summer^10^,^11^. Presently, white-plague type-II is among the most ubiquitous disease in the wider Caribbean, affecting up to 41 scleractinian coral species^13^,^14^.

Typically, white-plague disease is manifested as a rapidly expanding white ring of necrotic tissue on an otherwise healthy coral colony. White-plague disease generally commences at the base of the infected colonies, or from blemishes and lesions on the colony’s surface. Multiple diseased areas are often observed on the same colony. In most cases, white-plague disease radiates outward killing the coral, leaving behind bare white skeleton, which is subsequently colonized by turf algae or covered by sediment. The outward signs reported in the present study are similar to the pathologies of the white-plague type-II outbreak that were reported in 1995 on reefs in the upper Florida Keys^10^,^11^,^12^.

The putative pathogen of white plague type-II was isolated by Richardson *et al*.^11^, and was classified as a novel *Sphingomonas* bacteria. In the US Virgin Islands, analysis of tissue from five coral species confirmed the presence of a *Sphingomonas*-like bacterium in white-plague disease infected colonies^15^. More recently, the putative pathogen was identified as *Aurantimonas coralicida*, a newly described bacteria^16^. However, recent work has not detected this pathogen in coral samples with signs of the disease, indicating that there is probably more than one causative agent for white-plague disease^17^,^18^,^19^. Furthermore, the lack of host specificity, and the occurrence of similar symptoms with different microbial communities, led Lesser *et al*.^20^ to suggest that white-plague disease is simply an opportunistic infection in a compromised coral host. Moreover, the disease may be caused by a number of pathogens and environmental circumstances, culminating in the same physical signs that we presently identify as white-plague disease. Many coral reefs show low (<5%), year round, prevalence of white-plague disease^7^,^21^, suggesting that a ‘reservoir’ of white-plague disease might remain within coral populations in the Caribbean^12^,^15^. In general, even during active outbreaks, disease prevalence is often quite low (<10%)^21^,^22^,^23^,^24^. Despite the generally low prevalence, white-plague type-II is considered to be one of the most lethal coral diseases^12^,^22^,^25^,^26^,^27^,^28^,^29^.

Here we document the impact of a white-plague-disease outbreak on coral communities in Miami-Dade County, Florida. In the summer of 2014, numerous alerts were issued in response to anomalously warm sea surface temperatures in Florida^30^. By the late summer and early fall, many corals off southeast Florida expelled their symbionts in response to prolonged warm temperatures, resulting in the worst bleaching episode since 1997–1998, with corals, zoanthids, and octocorals all showing outward signs of stress^2^,^30^. In addition to extensive coral bleaching, there were numerous reports of coral disease throughout the Florida Keys^2^,^30^. The objectives of this study were to: (1) quantify the prevalence of white-plague disease on 14 reef sites in southeastern Florida (Fig. 1); (2) examine the timing of the onset of the disease outbreak at those sites; (3) determine whether there was any spatial pattern in the disease outbreak; and (4) quantify the overall impacts of the disease throughout the region.

## Results

### Tagged-coral colonies

**Figure 1:**
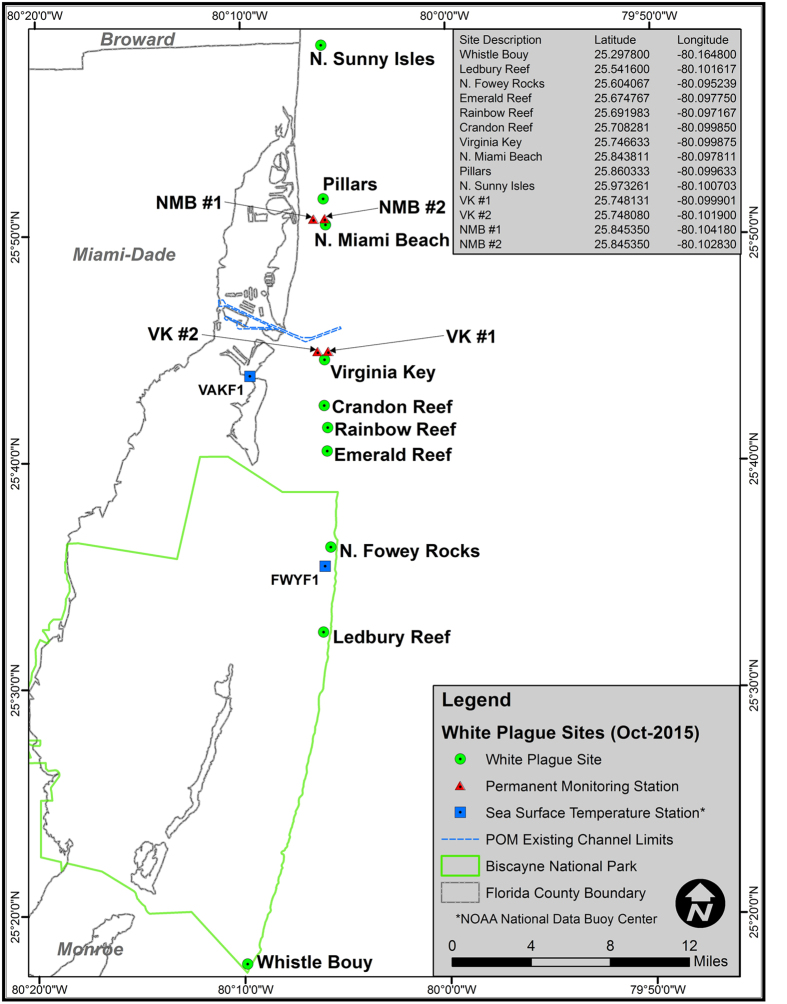
Location map of the 10 timed-swim sites (green dots) and four permanent coral monitoring sites (red triangles) off Miami-Dade County in southeast Florida. Location of the NOAA National Data Buoy Center sea surface temperature monitoring stations, VAKF1 (Bear Cut) and FWYF1 (Fowey Rocks) are denoted by blue squares.

**Figure 2:**
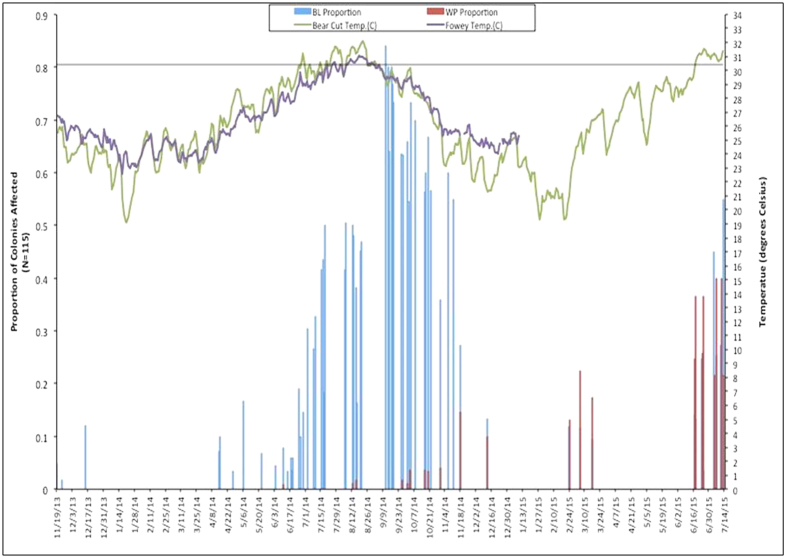
Graph showing the relationship between Sea Surface Temperature and prevalence of coral bleaching and white-plague disease at our four long-term monitoring stations. Temperature was downloaded from NOAA National Data Buoy Center, NOAA National Data Buoy Center, Virginia Key Station (VAKF1)^61^, located off the southern tip of Virginia Key in Bear Cut, and Fowey Rock Station (FWYF1)^62^, located due east of Soldier Key within Biscayne National Park. Fowey Rock data is depicted by solid purple line and Virginia Key “Bear Cut” Station by solid green line. Note the dampened temperature profile of the offshore Fowey Rock Station as compared with the inshore site from Bear Cut. Horizontal line across the graph at 30.4 °C represents regional coral bleaching threshold^2^. Coral bleaching prevalence, shown as blue histograms, is the proportion of paled and bleached tagged coral colonies recorded at the four long-term monitoring sites during weekly surveys. Similarly, white-plague disease prevalence, shown as red histograms, is the proportion of actively diseased tagged corals at these same sites.

**Figure 3:**
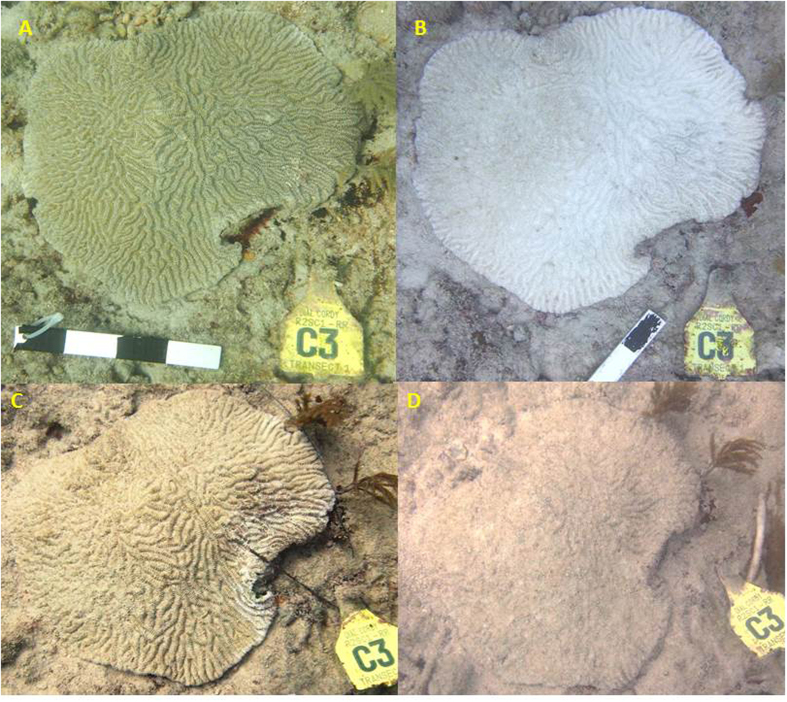
Time-series photographs of tagged *Meandrina meandrites* colony showing rapid impacts from bleaching and white-plague disease at one of the permanent monitoring sites off Virginia Key. Photo of unbleached living colony on August 20, 2014 (**A**); photo of same colony four weeks later during peak of coral bleaching event, September 23, 2014 (**B**); photo showing colony-wide recovery of zooxanthellae associated with seasonal drop in water temperatures, note residual “paled” coloration of coral tissue and first outward signs of white plague-like symptoms appearing along colony edge on November 11, 2014 (**C**); photo of same colony, totally dead from white-plague disease only four weeks later now covered with sediment laden algal turf, December 9, 2014 (**D**). Photos taken by RF.

A total of 115 coral colonies, representing 13 coral species, were tagged for repeated monitoring at four sites on the inner reef tract in Miami-Dade County (Fig. 1). Coral colonies were monitored at least 40 times between October 19, 2013 and July 13, 2015^31^. The peak of coral bleaching was recorded in September 2014. Specifically, the highest recorded bleaching prevalence occurred on September 12, 2014, when 84% (21 of 25 corals surveyed that day) showed signs of coral bleaching (Fig. 2). Recovery from bleaching was apparent soon after the temperatures decreased, with most corals regaining their color within 2 months (Figs 2 and 3). Only one tagged colony, in the four permanent monitoring sites, died as a direct result of coral bleaching.

The first sign of a white-plague disease outbreak (prevalence >5%) was noticed at the southern monitoring sites, near Virginia Key, on September 26, 2014 and by November 18, 15% of the tagged colonies showed outward signs of white-plague disease (Fig. 2). White-plague disease did not impact the northern monitoring sites (14 km north) until June 2015. During the week of June 17, 17% of the tagged coral colonies at the northern monitoring sites were infected with white-plague disease. In total, white-plague disease was observed on seven of the 13 coral species identified at the four permanent monitoring sites (Table 1). The highest recorded prevalence of white-plague disease occurred on July 7, 2015, when 40% (22 of 55 corals surveyed that day) showed signs of white-plague disease infection (Fig. 2). Of the white-plague impacted species, the overall disease prevalence was 51% (35 of 69 corals surveyed). The majority (81%) of white-plague susceptible corals bleached, prior to becoming infected with the disease (Table 1). *Meandrina meandrites, Dichocoenia stokesi, Colpophyllia natans*, and *Pseudodiploria strigosa* were the most heavily impacted coral species, with 100% of tagged colonies being afflicted with white-plague disease. All colonies that showed signs of disease died, regardless of species. The corals *Porites astreoides, Siderastrea siderea,* and *Stephanocoenia intercepta* were not affected by white-plague disease at our permanent monitoring stations (Table 1).

### Timed-swim surveys

**Figure 4:**
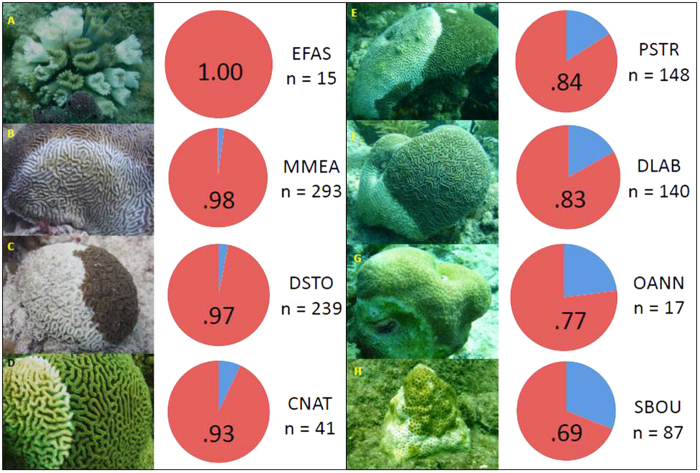
Photographs of the eight most affected corals to the recent white-plague epizootic off Miami-Dade County, FL, showing active disease on each species. Disease prevalence recorded is calculated from time-swim survey data (Fig. 1) and graphically represented as proportions, where red represents disease prevalence, and blue represents the proportion of colonies with no signs of disease. Disease prevalence includes colonies with both active signs of white-plague disease and those that were identified as recently killed as a direct result of the disease. The strong coupling between disease prevalence and total coral mortality suggests that disease prevalence was a useful proxy of mortality. Sample size (n=) is the total number of colonies observed at the ten (10) timed-swim survey sites. The combined coral disease prevalence for these eight species is 90%. Coral species abbreviations are as follows: *Eusmilia fastigiata* (EFAS), *Meandrina meandrites* (MMEA), *Dichocoenia stokesi* (DSTO), *Colpophyllia natans* (CNAT), *Pseudodiploria strigosa* (PSTR*), Diploria labyrinthiformis* (DLAB), *Orbicella annularis* (OANN), and *Solenastrea bournoni* (SBOU). Photographs taken by WFP.

A total of 21 coral species were recorded at the 10 timed-swim survey sites (Fig. 1). Active white-plague disease was observed on 12 of those coral species. The average prevalence of disease for all 21 species was 67% (441 of the 660 living corals surveyed). Of the 12 white-plague impacted species, the overall disease prevalence was 79% (441 of the 556 corals surveyed). However, when diseased and recently dead colonies were combined into a single category, disease prevalence increased to 81% (951 of the 1,170 corals surveyed; Table 2). At the sites surveyed *Eusmilia fastigiata, Dichocoenia stokesi, Meandrina meandrites, Colpophyllia natans, Diploria labyrinthiformis, Pseudodiploria strigosa*, and *Orbicella annularis* experienced the highest disease prevalence (these seven species had an average combined disease prevalence of 92%; see Table 2 and Fig. 4). Follow-up surveys, at two of the 10 sites, revealed that most colonies that were afflicted with white-plague disease had subsequently died. The corals *Porites porites, P. astreoides, Siderastrea siderea,* and *Stephanocoenia intersepta* did not show signs of white-plague disease during our surveys (Table 2). Preliminary reports from other studies performed throughout southeast Florida during 2014–2015 mirror the results from our surveys^31^,^32^.

### Disease spread

**Figure 5:**
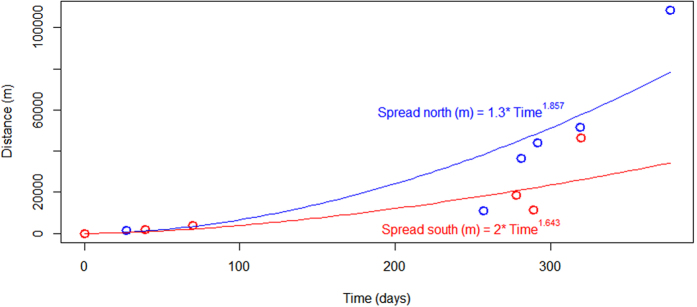
The best-fit function that described the relationship between the duration of time it took the disease to spread across reefs (in meters) is Distance (m) = 0.22.time in days^2.1399^ to spread north, and Distance (m) = 1.28.time in days^1.714^ to spread south. The circles in blue and red are the data indicating the first known arrival of the disease (see Table 3), with red depicting data south of Virginia Key, and blue circles depicting data north of Virginia Key.

The recent outbreak of white-plague disease was first observed in southeastern Florida in September, 2014. Mapping the relationship between the time of the first outbreak of disease and the geographic distance of potential spread, we noticed that the disease radiated out from Virginia Key, spreading both north and south. By August 12, 2015, the outbreak had been recorded from Whistle Buoy Reef in Biscayne National Park, in the south, to the Breakers Reef, off Palm Beach County, in the north (Table 3), a distance of approximately 130 km. However, the spread of the disease was more than three times greater to the north than it was to the south (Fig. 5).

## Discussion

The recent white-plague disease outbreak in southeastern Florida is unprecedented in terms of the total proportion of coral colonies infected, the high within species disease prevalence, and the extent of coral mortality. Field observations from our 14 study sites revealed that white-plague disease affected 61% of all coral species that were surveyed. However, when white-plague disease afflicted species were evaluated separately from the total coral population the disease prevalence increased to 79%. When recently dead corals were added to the totals, the prevalence of white-plague disease increased to 81%. These diseased corals, in-turn, had the highest estimate of coral disease prevalence on record, with many species exceeding 90%. One hundred percent of the surveyed colonies of *E. fastigiata* were infected with white-plague disease, or had recently been killed by the disease. Repeat monitoring of tagged colonies showed that all cases of colonies with white-plague infection led to total colony mortality, regardless of the species infected. The strong coupling between disease prevalence and total coral mortality suggests that disease prevalence was a useful proxy of mortality. The most heavily impacted corals were *Eusmilia fastigiata, Meandrina meandrites,* and *Dichocoenia stokesi,* showing regional losses of >97% of the coral colonies. In addition, a number of other coral species *Colpophyllia natans, Pseudodiploria strigosa, Diploria labyrinthiformis,* and *Orbicella annularis*, were reduced to <25% of the initial population densities. The long-term ecological persistence of these species, throughout southeast Florida, is questionable following such high mortality.

Previously, the highest recorded levels of disease-related coral mortality were associated with white-band disease^6^. From the late 1970 s through the 1990 s, numerous and often repetitive white-band disease outbreaks were responsible for reducing acroporid populations by >95% across the Caribbean^6^. The greatest recorded impact of a white-plague disease outbreak was ~60% loss of coral cover in the US Virgin Islands following the 2005 thermal anomaly^28^,^33^. The 2014-2015 outbreak of white-plague disease in southeast Florida caused considerably greater within species losses than the 2005 event, and impacted a total of at least 13 coral species. Based on the number of coral species affected, the high prevalence of disease, and extent of coral mortality the 2014-2015 outbreak is arguably one of the most lethal coral-disease outbreaks ever recorded on a contemporary coral reef.

The unprecedented disease outbreak in 2014 to 2015 appears to have been associated with the unusually warm-winter and spring temperatures^2^,^30^, followed by an anomalously warm summer. These conditions caused widespread bleaching, which was most likely a precursor of the lethal white-plague outbreak that followed (see Fig. 2). There is increasing evidence that corals that have been heat stressed are more susceptible to disease than corals that have not been heat stressed^34^. Recently climate change has exacerbated the severity of coral diseases, and the links between temperature and disease outbreaks continue to escalate^4^,^5^,^24^,^33^,^34^,^35^,^36^.

A link between thermal stress and the onset of a white-plague disease outbreak has been previously reported. A white-plague disease outbreak was reported immediately following the 2005 bleaching event, showing high levels of coral mortality on many reefs throughout the Caribbean^27^,^28^,^29^,^33^,^35^,^37^. Muller *et al*.^35^ showed that the prevalence of coral disease increased following the 2005 thermal-stress event, but they also showed that disease-associated mortality was only apparent when the coral host had bleached prior to the disease outbreak. In a similar study, Brandt and McManus^37^ documented that colonies with white-plague disease experienced the most extensive bleaching during the 2005 thermal anomaly. Together, these results reflect a clear and consistent relationship between bleaching and disease prevalence (see also^29^). The results of the 2014–2015 white-plague outbreak are similar, but in the present study bleaching was not a prerequisite of infection (Table 1). Yet, outbreak levels (>5% prevalence) of white-plague disease were not documented until after the peak of the thermal stress event in 2014 (Fig. 2). The established pattern of high prevalence of white-plague disease following thermal anomalies suggests that thermal-stress events are the seasonal drivers of white-plague disease outbreaks, and that coral bleaching most likely enhances the severity of the disease. The above interpretation also suggests that the impact of coral diseases will increase with future global warming^4^,^38^.

Despite links between thermal stress and coral disease, it is unknown whether or not coral diseases in general, and white-plague disease in particular, are contagious, or whether the observed diseases are merely secondary responses to environmental stress. The 2014–2015 white-plague disease outbreak clearly follows a contagion-based model. Both the 2014-2015 outbreak of white-plague disease and the outbreak recorded in 1995, in the upper Florida Keys, radiated from a ‘point’ of origin^11^. Sokolow *et al*.^39^ showed that there was a good fit between metapopulation epidemiological models, and the empirical data for the outbreak in 1995 in upper Florida Keys. The current outbreak appeared to originate near Key Biscayne/Virginia Key (Table 3). The rates of spread of white-plague disease were more rapid to the north than to the south (Fig. 5). These differences were likely a consequence of the northerly flow of the Florida Current. It is therefore not surprising that the southerly spread occurred more slowly than the northerly spread. There are, however, south flowing coastal counter-currents, which could explain the southward advection, albeit more slowly than to the north. Similarly, Lessios *et al*.^40^ showed that the Caribbean-wide pandemic of the long-spined sea-urchin, *Diadema antillarum,* in 1983–1984, did not always follow the direction of the major north-flowing currents off southeast Florida and the Florida Keys. In addition, the white-plague outbreak in the Florida Keys in 1995–1997 similarly did not always follow the direction of the major north-flowing currents^11^. The transmissibility of white-plague disease strongly implies that this particular strain is water-borne, infectious, and highly contagious.

Despite potentially similar modes of transmission between the 1995 and the 2014–2015 white-plague disease outbreaks, the more recent outbreak is unprecedented, in that it spread throughout an impoverished coral community with less than 3% coral cover. It is well known that the potential transmission of a disease is influenced by the density of its hosts^4^,^34^,^36^,^39^,^40^,^41^. Indeed, Bruno *et al*.^34^ showed that infection rate of coral diseases should increase linearly with population size (see also^41^), and therefore, infection rate should be relatively low in a population with a low density. However, the low coral population densities observed off Miami-Dade County in particular, and the rest of the southeast Florida reef tract in general, were not immune to this outbreak of white-plague disease^31^,^32^. The results also suggest that assemblages of low-density coral populations will not necessarily be safe havens from future epizootic episodes.

Cróquer *et al*.^42^ suggested that white-plague disease may ultimately have a greater impact on the Caribbean than other diseases, including white-band disease. The increasing records of white-plague outbreaks following major coral thermal-stress events^24^,^25^,^26^,^27^,^28^,^29^,^33^, and the sudden decline of entire populations of several coral species in southeastern Florida, suggests that white-plague disease is already having a major impact on the coral populations of the Caribbean. In fact, modeling forecasts of white-plague disease in the Red Sea show that for some global warming scenarios, even an increase of only 0.5 °C in sea surface temperature can cause epidemics to double in magnitude^38^, portending additional decline of reef-coral populations in the Caribbean associated with global climate change. Ultimately, the coral species with the greatest resistance, with the ability to acclimate, and with the capacity to adapt to temperature stress and disease will dominate reef communities in the future^43^,^44^,^45^. This ’filtering’ of species may already be occurring in many places throughout the Caribbean, resulting in novel communities^43^,^44^,^45^,^46^. For example, in Florida we have been witnessing a rise in relative dominance of *Siderastrea siderea* and *Porites astreoides*^43^,^45^,^47^,^48^. These two coral species were not affected by the recent white-plague disease event, and recently show increasing densities.

The documentation of the 2014–2015 white-plague outbreak in southeastern Florida is a dramatic example of how coral bleaching and disease can irrevocably change coral populations within a short period of time. The high prevalence and transmissibility of the disease through coral communities with low densities, and against predominant currents suggest that all coral communities, regardless of how depauperate or remote, may be affected by climate-mediated disease outbreaks. Unfortunately, there is little that local resource managers can do to stop a thermal-stress event, or stop a disease outbreak, or change the overall trajectory of coral loss associated with regional and global disturbances^49^,^50^. Removing or ameliorating local causes of coral mortality is an important goal of management; however, effective local management alone is not sufficient to stem the decline of coral populations at the regional and global level. These thermal events highlight the need to focus on conservation efforts that curb atmospheric CO_2_ emissions^51^,^52^. At best, failure to do so will likely result in novel coral communities, comprised of only the hardiest and weediest of species^43^,^44^,^45^,^47^,^48^, which tolerate not only ocean warming but also coral diseases^2^,^43^,^49^. For some locations, such as the reefs in southeast Florida, this community shift is already underway^45^,^48^,^49^,^50^. At worst, as global climate change continues, chronic, thermally-induced coral-mortality events will continue to increase in severity and recur more frequently. There is also an increased probability of coral extirpations and extinctions by the end of the century^51^,^53^,^54^. In either case, if the events of 2014–2015 in southeastern Florida are a bellwether for the future, then the coral communities of tomorrow will look and function differently from those of today.

## Methods

### Study Area

This study was undertaken on the coral reefs in southeastern Florida, which are at the northern extent of reef growth along America. Lying off Miami-Dade County are a series of submerged, shore-parallel, fossil reef terraces. These relict reefs are the remains of a nearly continuous, 150 km long, barrier-reef system that extended northward from Miami to Palm Beach County in the early to middle Holocene^50^,^55^. In Miami-Dade County the two main terraces are referred to as the inner (second) and outer (third) reefs; closer to shore a fossilized coquina beach deposit is apparent, also known as the first reef or nearshore ridge complex^55^. Modern-day assemblages of stony corals and octocorals have colonized these relict reefs and nearshore hardbottoms. The coral assemblages documented in this study are all from the inner (second) reef terrace. These assemblages have low species richness and low coral cover^56^,^57^,^58^. Scleractinian colony density along the inner reef terrace ranges from 0.95 to 2.49 colonies per square meter^31^. The average diameters of the coral colonies are generally small (<25 cm), with proportionally few colonies exceeding 50 cm diameter. In Miami-Dade County the inner reef terrace lies 1.5–2.0 km from shore in water depths ranging from 6–11 m. Small-scale latitudinal differences have been noted with an overall reduction in community diversity and coral cover from south to north^57^. For instance, coral cover on reefs south of Key Biscayne into Biscayne National Park averaged around 3% in the early 2000 s^59^,^60^. Coral cover on the inner reef tract off Miami-Dade County, north of Key Biscayne is routinely measured at <0.05%^57^,^58^.

### Tagged Coral Surveys

Four permanent study sites were established in October 2013; two off Virginia Key and two off North Miami Beach. These four sites were originally designed as controls for environmental compliance monitoring associated with the Port Miami deepening project^31^ (Fig. 1). Water depth varied between 6–8 m at these sites. At each site, three permanent 20-meter transects were established parallel to each other. On each transect, a set of up to ten (10) stony coral colonies, within one meter from the line, were selected for monitoring. Because the density of scleractinian coral colonies was low throughout the study area, adjustments to exact transect placement in the field were conducted to avoid sandy areas, to maximize hardbottom, and to maximize the number of hard corals sampled along each transect. Each coral colony that was selected for monitoring was subsequently marked with a numbered plastic tag, attached to the hardbottom, next to the colony (Fig. 3). *In situ* coral-condition data were collected by scientific divers for all tagged scleractinian species. Still photographs of each of the permanently marked corals were taken in planar view, so that each colony was present within a single photo frame, along with the permanent marker and scale bar (Fig. 3). Tagged corals were photographed using an Olympus TEN micro 4/3 digital camera with a 9-mm wide-angle lens and underwater housing with external strobe lighting. A total of 115 healthy coral colonies were initially tagged within the four study sites. Most tagged corals were small, ranging from <10–40 cm in diameter. Between October 19, 2013 and July 13, 2015 each tagged coral, at each site, was monitored and photographed at least 40 times^31^. In the laboratory, >5,000 individual observations of *in situ* coral condition were compared to paired photographs. From these data we were able to calculate the prevalence of both coral bleaching and disease at various snapshots in time (Fig. 2). We define white-plague prevalence as the proportion of cases that were present in the population, at a given time. In addition, in cases where corals had died, we were generally able to discern the exact cause of mortality by carefully evaluating the sequence of events recorded (and photographed), prior to death.

### Timed Swim Surveys

We used timed swims at ten sites along the inner-reef tract that were geographically widespread throughout the waters of Miami-Dade County (Fig. 1). The surveys were performed between November 2014 and August 2015. The timed-swim surveys documented disease prevalence and attempted to track the regional spread of the disease outbreak. Each timed swim was 60 minutes in duration. The diver entered the water at a fixed geographic coordinate and swam in a roughly rectangular pattern, so as to return to the same point at the end of the dive. Hypack Navigational ™ software was used to record the geographic location of the starting point for each of the ten survey sites. Water depth varied between 6 m and 11 m at these sites. At each site, the diver slowly swam at a height of approximately 1 m above the substrate, stopping occasionally to take notes and photographs. Approximately 300 m^2^ of reef benthos was sampled during each survey. The diver recorded the total number of all coral colonies visually encountered, and each colony was identified to species level. Notes were taken on their condition (i.e., healthy, bleached, diseased, or recently dead) on an underwater tablet; coral colonies <5 cm in diameter were not included in the surveys. Recent mortality was designated to colonies with exposed white skeleton, with minimal algal turf. In addition, corals living in cryptic environments, and not easily seen from above, were not included in the surveys. Corals that were bleached, visibly diseased, or recently dead during the surveys were photographed using a Sealife DC600 underwater camera and housing. Follow-up surveys were undertaken on Rainbow Reef and North Sunny Isles Reef on September 19–20, 2015. These surveys examined whether the disease was still active, and divers recorded the general state of the coral communities at these sites.

### Seawater Temperatures

We used sea surface temperature data from the NOAA National Data Buoy Center, Virginia Key Station (VAKF1)^61^, located off the southern tip of Virginia Key in Bear Cut, and Fowey Rock Station (FWYF1)^62^, located due east of Soldier Key within Biscayne National Park (Fig. 1). These data were used for temporal comparison with coral bleaching and disease prevalence data recorded at our permanent coral monitoring sites (Fig. 2). The Fowey Rock station is part of the SEAKEYS network of automated monitoring stations installed upon navigational structures situated along the length of the Florida reef tract. These stations transmit hourly data of mean wind speed, wind direction, and depth-averaged *in-situ* sea temperature in standard C-MAN format, via NOAA geosynchronous operational satellites.

### Contagion Spread

The relationship between the date of first records of white-plague disease outbreak and the geographic distance from Virginia Key, where the white-plague disease outbreak was first recorded (Table 3), were first examined graphically, and then subsequently fitted to an exponential function using the packages ‘nls’ in R^63^.

## Additional Information

**How to cite this article**: Precht, W. F. *et al*. Unprecedented Disease-Related Coral Mortality in Southeastern Florida. *Sci. Rep.* **6**, 31374; doi: 10.1038/srep31374 (2016).

## Figures and Tables

**Table 1:** Combined data for all tagged corals at the four permanent monitoring stations (Fig. 1), where BL indicates bleached, and WP indicates white-plague.

Species	Colonies (n)	BL Colonies	% BL	WP Infected Colonies	% WP
*Dichocoenia stokesi*	10	4	40	10	100
*Meandrina meandrites*	9	8	89	9	100
*Pseudodiploria strigosa*	4	3	75	4	100
*Colpophyllia natans*	1	1	100	1	100
*Montastraea cavernosa*	27	24	89	8	29
*Pseudodiploria clivosa*	6	5	83	1	16
*Solenastrea bournoni*	12	11	92	2	16
*Siderastrea siderea*	17	14	82	0	0
*Porites astreoides*	17	14	82	0	0
*Stephanocoenia intersepta*	5	5	100	0	0
*Acropora cervicornis*	4	2	50	0	0
*Orbicella faveolata*	2	0	0	0	0
*Agaricia agaricites*	1	1	100	0	0
**Total**	115	92	80	35	30

Total corals, n = 115.

**Table 2:** Combined data for all corals observed at the ten (10), timed-swim survey sites.

Species	Total colonies (Live + DIP) (n)	Total Live Colonies (n)	Healthy	WP Infected	% WP (Live Only)	DIP	Combined WP & DIP	% Combined
*Eusmilia fastigiata*	15	9	0	9	100	6	15	100
*Meandrina meandrites*	293	75	6	64	85	223	287	98
*Dichocoenia stokesi*	239	53	6	47	89	186	233	97
*Colpophyllia natans*	41	25	3	22	88	16	38	93
*Pseudodiploria strigosa*	148	122	24	98	80	26	124	84
*Diploria labyrinthiformis*	140	112	24	88	79	28	116	83
*Orbicella annularis*	17	15	4	11	73	2	13	77
*Solenastrea bournoni*	87	75	27	48	64	12	60	69
*Occulina diffusa*	2	2	1	1	50	0	1	50
*Montastraea cavernosa*	155	145	96	49	34	10	59	38
*Mycetophyllia aliciae*	3	2	2	0	0	1	1	33
*Orbicella faveolata*	30	30	26	4	13	0	4	13
*Stephanocoenia intercepta*	60	60	60	0	0	0	0	0
*Siderastrea siderea*	244	24	244	0	0	0	0	0
*Porites astreoides*	257	257	257	0	0	0	0	0
*Porites porites*	18	18	18	0	0	0	0	0
*Agaricia agaricites*	39	39	39	0	0	0	0	0
*Agaricia lamarcki*	8	8	8	0	0	0	0	0
*Madracis decactis*	4	4	4	0	0	0	0	0
*Acropora cervicornis*	(66)	(66)	(24)	—	—	—	—	—
**Totals (with and without** ***A. cervicornis***)	**1896/1830**	**1141/1075**	**902/878**	**441/441**	**38/41**	**508/508**	**951/951**	**50/52**

Total number of colonies is shown with and without *A. cervicornis*. White-plague prevalence calculations omit *A. cervicornis* data as this is not a white-plague susceptible species *sensu* Weil^13^, where BL indicates bleached, WP indicates white-plague, and DIP indicates recently dead in-place. % Combined column includes colonies with both active signs of white-plague disease and those that were identified as recently killed as a direct result of the disease.

**Table 3:** Locations of reef sites used in calculations of disease spread.

Site	Lat	Long	First Reported Outbreak	Reference
Whistle Buoy	25° 17′ 52.08″	80° 09′ 53.28″	8/12/2015	W. Precht
Emerald Reef	25° 40′ 29.16″	80° 05′ 51.90″	12/5/2014	P. Jones
Rainbow Reef	25° 41′ 31.14″	80° 05′ 49.80	11/4/2014	J. Shekels
Virginia Key	25° 44′ 47.88″	80° 05′ 59.55″	9/26/2014	DCA^31^
Government Cut	25° 45′ 36.05″	80° 05′ 59.27″	10/23/2014	DCA^31^
N. Miami Beach	25° 50′ 37.72″	80° 05′ 52.12″	2/24/2014	DCA^31^
N. Sunny Isles	25° 58′ 23.74″	80° 06′ 02.53″	6/12/2014	W. Precht
Hollywood–Broward	26° 04′ 15.18″	80° 05′ 45.05″	7/4/2015	W. Precht
Sunrise – Broward	26° 08′ 22.32″	80° 05′ 38.07″	7/15/2015	D. Clark
Lauderdale by the Sea – Broward	26° 12′ 20.04″	80° 05′ 06.47″	8/11/2015	E. Peters
Breakers Reef - Palm Beach	26° 42′ 48.09″	80° 01′ 47.99″	9/13/2015	K. Bohnsack

Date of first arrival is based on data collected from a number of sources including personal observations. Locations are listed sequentially from south to north and extend from southernmost Miami-Dade County to central Palm Beach County, a distance of approximately 130 km.
